# Developing Biomarkers for the Skin: Biomarkers for the Diagnosis and Prediction of Treatment Outcomes of Alzheimer’s Disease

**DOI:** 10.3390/ijms24108478

**Published:** 2023-05-09

**Authors:** Ching-Ying Wu, Chih-Yi Ho, Yuan-Han Yang

**Affiliations:** 1 Department of Dermatology, College of Medicine and Post Baccalaureat Medicine, Kaohsiung Municipal Ta-Tung Hospital, Kaohsiung Medical University Hospital, Kaohsiung Medical University, Kaohsiung 807, Taiwan; dermacyw@kmu.edu.tw (C.-Y.W.);; 2 Department of Cosmetic Science, Chang Gung University of Science and Technology, Taoyuan 333, Taiwan; 3 Department of Neurology, College of Post Baccalaureat Medicine, Kaohsiung Municipal Ta-Tung Hospital, Kaohsiung Medical University Hospital, Kaohsiung Medical University, Kaohsiung 807, Taiwan

**Keywords:** Alzheimer’s disease (AD), biomarker, skin physiological test, skin hydration, pH level, elasticity, capillary microscopy, non-invasive diagnosis, capillary tortuosity, ApoE genotype, prognosis

## Abstract

Alzheimer’s disease (AD) is a neurodegenerative disorder characterized by memory decline and cognitive impairment. Research on biomarkers can aid in early diagnosis, monitoring disease progression, evaluating treatment efficacy, and advancing fundamental research. We conducted a cross-sectional longitudinal study to see if there is an association between AD patients and age-matched healthy controls for their physiologic skin characteristics, such as pH, hydration, transepidermal water loss (TEWL), elasticity, microcirculation, and ApoE genotyping. The study used the Mini-Mental State Examination (MMSE) and Clinical Dementia Rating-Sum of the Boxes (CDR-SB) scales as references to quantify the presence of disease, if any. Our findings demonstrate that AD patients have a dominantly neutral pH, greater skin hydration, and less elasticity compared to the control subjects. At baseline, the tortuous capillary percentage negatively correlated with MMSE scores in AD patients. However, AD patients who carry the ApoE E4 allele and exhibit a high percentage of tortuous capillaries and capillary tortuous numbers have shown better treatment outcomes at six months. Therefore, we believe that physiologic skin testing is a rapid and effective way to screen, monitor progression, and ultimately guide the most appropriate treatment for AD patients.

## 1. Introduction

Dementing illnesses such as Alzheimer’s disease (AD) are significant health problems in the aging population. The prevalence of clinically diagnosed AD dementia rises with increasing age. Early detection of AD is challenging, and it is estimated that AD affects approximately 4% of adults in their 60s or late-stage employment [1]. Current research and treatments for AD have moved toward very mild stages in order to potentially provide effective therapy. Therefore, how to detect very mild dementia is becoming an important issue, especially with the aid of biomarkers, which can improve diagnostic accuracy. Although the DSM-1V was considered the golden standard for diagnosing AD, its pathologically validated accuracy was only approximately 80% [2]. The search for a biological marker that can predict or confirm AD is an important area of research. Among the biomarker technologies that are currently under development are in vivo brain imaging, including imaging of amyloid plaques using the PET-Pittsburgh compound [3], molecular measures in cerebrospinal fluid (CSF) [4], and a variety of biochemical measures in peripheral tissues [5,6].

Direct measures of amyloid plaques, tau protein, beta-amyloid 1–40, and/or beta-amyloid 1–42 in the CSF may increase diagnostic accuracy. However, they may not lend themselves to the diagnostic screening of many patients, given the invasiveness of the required lumbar puncture procedure, which is remarkably more invasive than skin physiological measurement. Furthermore, some of the reported results have not shown consistency between laboratories [7,8]. The lumber puncture procedure is undoubtedly invasive and cannot be performed in routine AD evaluation, although it is a standard feature of dementia investigation in many countries. Nevertheless, a peripheral biomarker would be more convenient for clinicians and patients. For useful screening of an elderly population, the biomarker should be non-invasive and relatively less expensive.

Recently, there has been increasing evidence that the brain and skin are intertwined more closely than expected. Beta-amyloid may deposit in the brain, leading to the diagnosis of AD, and it may also deposit in the skin, especially in fibroblasts, leading to changes in cutaneous micro- and macrovascular function [9,10].

In the physiological view of skin, Presenilin-1 is a catalytic subunit of a protease that cleaves APP (amyloid precursor protein) and is involved in epidermal growth factor receptor (EGFR) turnover. Partial loss of presenilin-1 expression has been proven to lead to seborrheic keratoses and inflammatory skin diseases [11,12]. Antioxidant defenses in skin fibroblasts from patients with familial AD have been shown to be lower compared to controls [13]. Skin fibroblasts from patients with AD exhibit dysfunctional bradykinin receptor signaling [14] and altered cholesterol ester metabolism [15] as well as enhanced GMI ganglioside catabolism [16]. Calcium homeostasis and mitochondrial function are also altered in AD skin fibroblasts [17]. In the skin, mastocytes can express beta-amyloid and tau-protein in patients with AD, which may be related in part to the inflammation secondary to epidermal keratinization, changes in pH value, and transepidermal water loss [18,19,20,21,22].

These growing pieces of evidence have shown a close connection between the brain and skin in AD. Therefore, it is plausible that the skin may provide benefits for the early diagnosis of AD, increase diagnostic accuracy, and serve as a biomarker to trace the clinical course of AD. This study aims to demonstrate that skin can be beneficial for the early diagnosis of AD, increasing diagnostic accuracy, and serving as a biomarker to trace and reflect the clinical course of AD.

## 2. Results

### 2.1. AD Patients Have a Higher Skin PH Level, Higher Hydration, and Lower Elasticity Than Healthy Individuals

This study included 29 patients with AD and 12 healthy controls (Table 1). The proportion of females in the case and control groups was 75.86% and 75.00%, respectively, with mean ages of 78.72 ± 7.319 years and 75.33 ± 4.459 years, respectively. We also measured skin physiological values, including skin pH, hydration, elasticity, capillary flow rate, total capillaries, tortuous capillary number, and percentage of tortuous capillaries. Compared with healthy controls, AD patients had a more neutral skin pH (6.541 ± 1.357 vs. 5.900 ± 0.641, *p* = 0.0294), higher skin hydration values (90.52 ± 12.59 vs. 73.50 ± 15.09, *p* = 0.0012), and significantly decreased average elasticity values than the control group (48.48 ± 15.15 vs. 62.33 ± 7.09, *p* = 0.0019).

### 2.2. AD Patients’ Percentage of Tortuous Capillaries Is Negatively Correlated with Their Cognitive Performance at Baseline

In the assessment of AD, the Mini-Mental State Examination (MMSE) and Clinical Dementia Rating-Sum of the Boxes (CDR-SB) are two essential scales. In Table 2, we investigated the correlation between the scores of these two scales and skin physiological values in AD patients at baseline. There was no statistically significant correlation between MMSE and CDR-SB scores (with an R-value of −0.318; *p* = 0.092). The analysis showed a statistically significant negative correlation between the percentage of tortuous capillaries and MMSE score (with an R-value of −0.49, 95% confidence interval of −0.73 to −0.14, and a *p*-value of 0.0066). However, this study did not show a statistically significant correlation between CDR-SB scores and skin physiological values. See Supplement Figures S1 and S2 for details.

### 2.3. Higher Numbers of Tortuous Capillaries Are Associated with Better Treatment Responses in AD Patients

To investigate the relationship between therapeutic effect and skin physiological parameters in AD patients, we evaluated changes in MMSE and CDR-SB scores after six months of treatment with a cholinesterase inhibitor. Patients who showed an improvement, defined as a post-treatment MMSE score minus the pre-treatment score of ≥0, were categorized as responders, while those with a difference of <0 were categorized as non-responders. Of the 29 patients evaluated, 14 were responders and 15 were non-responders. We compared various skin physiological parameters between these two groups and found that responders had a higher percentage of tortuous capillaries than non-responders, with values of 0.52 ± 0.22% and 0.35 ± 0.14%, respectively, and a statistically significant difference (*p* = 0.022), as shown in Table 3.

We also evaluated the therapeutic effect using the CDR-SB, which indicates greater disease severity with higher scores. Patients with a post-treatment score minus a pre-treatment score ≤0 were categorized as responders, while those with a difference > 0 were categorized as non-responders. Among the 29 AD patients evaluated, 17 experienced a decrease in CDR-SB scores after treatment, while 12 showed no improvement. The responders had a higher number of tortuous capillaries than the non-responders, with values of 4.35 ± 1.66 and 3.08 ± 0.17, respectively, and a statistically significant difference between them (*p* = 0.046). Regarding the percentage of tortuous capillaries, the responders also had a higher ratio of tortuous capillaries than the non-responders (0.48 ± 0.21% vs. 0.36 ± 0.17%), but the difference was not statistically significant (*p* = 0.168), shown in Table 4.

### 2.4. A Higher Percentage of Tortuous Capillaries and the ApoE (E4) Genotype Is Associated with a Better Prognosis in AD Patients

We further conducted statistical analysis using logistic regression to evaluate the odds ratios (ORs) of age, gender, education level, ApoE type, and various physiological measurements (pH value, hydration, elasticity, capillary number, capillary flow rate, capillary tortuous number, percentage of tortuous capillary, and blood flow) versus the therapeutic efficacy of AD. Table 5 presents the results of the efficacy assessment using the MMSE scale after six months of treatment. We found that a higher percentage of tortuous capillaries was significantly positively correlated with the therapeutic efficacy, with an OR of 276.18 (*p* = 0.033). Carrying the ApoE E4 allele was also positively correlated with therapeutic efficacy, with an OR of 8.25, but this did not reach statistical significance (*p* = 0.073). The results of the efficacy assessment using the CDR-SB scale are presented in Table 6. We found that carrying the ApoE E4 allele was significantly positively correlated with therapeutic efficacy, with an OR of 13.00 (*p* = 0.031). Although the tortuous capillary number and percentage of tortuous capillaries were also positively correlated with therapeutic efficacy, with ORs of 1.70 (*p* = 0.056) and 34.49 (*p* = 0.125), respectively, they did not reach statistical significance.

## 3. Discussion

This study found that compared to healthy controls, the AD patient group had more neutral pH values and higher skin hydration values in terms of skin characteristics compared to controls. However, the average elasticity value of the patient group was statistically significantly lower than that of the control group. Additionally, in AD patients, the baseline microvascular tortuosity ratio was negatively correlated with MMSE scores. The post-treatment statistical results showed that, compared to the non-responder group, the responder group had a higher microvascular tortuosity ratio and microvascular tortuosity. Furthermore, carrying the ApoE E4 genotype was correlated with a more effective response to treatment. These data suggest that skin physiological parameters and a specific ApoE genotype can provide a certain degree of reference value in predicting the treatment outcomes of AD patients.

Although AD is known as a progressive neurodegenerative disease, an increasing body of research has confirmed that it is also a vascular disease with systemic effects [23,24,25]. As early as 2002, it was proposed that vascular pathology, including vascular damage, circulatory abnormalities, and cerebrovascular disease [23], are important characteristics of AD. The authors further proposed the “vasculopathy complex” to explain these phenomena, suggesting that vascular pathology may be one of the causes of AD and that treating hypertension and heart disease can slow down its development. The relationship between peripheral blood flow and AD has been a focus of research, and the interaction between the two is known to be complex. However, several factors may regulate this relationship. Blood flow may affect the severity of AD. Kashibayashi, T. et al. used single photon emission computed tomography (SPECT) to measure regional cerebral blood flow (CBF) in AD patients and found that decreased regional CBF was positively correlated with the severity of cognitive impairment [26]. The researchers also found that improving regional CBF could enhance cognitive function. Cooper, L.L. et al. examined the impact of atherosclerosis and cerebrovascular dysfunction on memory function [27] and suggested that atherosclerosis could affect CBF and perfusion pressure, leading to cerebrovascular dysfunction. This dysfunction could cause cognitive impairment and memory decline, increasing the risk of diseases such as AD. Certainly, increasing evidence has demonstrated that reduced blood flow might worsen AD; however, detecting CBF requires time-consuming clinical techniques and is expensive. More research is seeking faster and more convenient evaluation methods. Our study found that capillary tortuosity is related to AD severity and treatment response, and nail fold microcirculation can indirectly represent cerebral blood circulation due to the close connection between the peripheral and cerebral vascular systems. Smith et al. [28] reported a correlation between cognitive decline and disease severity in Alzheimer’s patients with whole blood viscosity and microvascular abnormalities. These factors can affect peripheral blood flow and vascular resistance, which in turn impact cerebral blood perfusion and metabolism. AD is associated with cerebral hypoperfusion, and vascular dysfunction may exacerbate disease severity [29]. The nail fold microvasculature is one of the smallest vessels in the human circulatory system, and its narrowness and tortuosity directly affect microcirculation permeability and blood flow velocity. AD patients exhibit microcirculatory abnormalities in the brain, which may also affect the function of nail fold capillaries.

Additionally, microvascular abnormalities in AD patients may affect the neuronal, glial, and capillary cells that maintain physiological balance in the brain, thereby impacting the stability and function of the neural network. A combination of these factors may lead to cognitive decline and increased disease severity in AD patients. Therefore, assessing the degree of tortuosity of nail fold capillaries or the blood flow velocity may indirectly reflect the cerebral microcirculation status of AD patients. Based on the results of previous studies, we can conclude that there is a complex interaction between peripheral blood flow and Alzheimer’s disease. Our study shows a correlation between the number of tortuous capillaries and favorable treatment outcomes. It is reasonable to assume that when the curvature of microvessels increases, medication can diffuse more into the target area due to slower blood flow and increased contact surface area between capillaries and tissues, resulting in better treatment response in Alzheimer’s disease patients who continue to receive medication.

Research using the skin as a medium to assist in diagnosing AD has been reported. Previous studies have found that Aβ protein also exists in the skin of AD patients, leading to abnormal fibroblasts in the skin [30,31,32]. However, these studies mainly focused on cellular-level changes and required special treatment of cells and molecular biology methods, which are time-consuming and costly. We used the Multi Skin Test Center MC900^®^ for skin physiological examinations and found that AD patients’ skin characteristics differed from those without the disease, including a neutral pH, higher hydration, and lower elasticity.

The normal pH of healthy skin is approximately 5.5, which indicates a slightly acidic environment. The skin is capable of self-regulating its pH in the presence of an intact skin barrier function, even when exposed to external factors that temporarily alter its pH, such as soap. In AD patients, the skin pH tends to be closer to neutral, suggesting impaired natural pH regulation or potential inflammation in the epidermis. Akerman et al. [33] confirmed the presence of inflammatory mediators, such as thymosin β-4 and psoriasin, in the epidermis of AD patients’ skin using mass spectrometry tissue imaging (MALDI-MSI) and skin tissue sections, which may interfere with the skin’s natural pH regulation function.

The higher hydration levels observed in the AD patients enrolled in this study were likely attributed to the better quality of care provided by their family members. These patients required frequent monitoring and follow-up for six months before and after treatment and could not often perform activities of daily living themselves, thus requiring assistance from family members and healthcare providers to attend hospital visits. Compared to the control group, the better performance in skin hydration levels among the enrolled patients might result from the support and care provided by their family members regarding home skin care.

The lower elasticity of the skin in AD patients may be indirectly related to the presence of Aβ protein in their skin, which affects fibroblasts. This study measured skin elasticity using the Multi Skin Test Center MC900^®^. When the probe is placed on the skin, it applies pressure to the skin surface. The tissue beneath the skin surface reacts to this pressure and disperses the energy into the surrounding tissue. The sensor in the probe detects this reaction, and the MC900 system converts the data into values of skin elasticity. The production and secretion of collagen, elastin fibers, and matrix molecules are mainly responsible for skin elasticity and resilience, and are mainly carried out by fibroblasts [34,35]. Therefore, skin elasticity is closely related to fibroblasts, and the function of fibroblasts may be affected by skin aging or other external factors, leading to a decrease in skin elasticity. Aβ protein is known to exist in the skin of AD patients and affects the function of fibroblasts, which may indirectly decrease skin elasticity in patients. Although limited studies have directly investigated the relationship between AD and skin elasticity, several physiological and pathological factors associated with AD may affect skin elasticity. Malnutrition is common amongst AD patients [36], including deficiencies in collagen and other proteins that may affect skin structure and elasticity. In addition, inflammatory biomarkers in the blood of AD patients are statistically significantly increased [31], which may affect the synthesis and secretion of collagen and elastin. Protein malnutrition and inflammatory diseases maximize the risk of neurodegenerative effects [37]. Furthermore, AD patients may reduce daily activity due to decreased motor ability, which may also affect skin elasticity. A study conducted by De la Rosa, A. et al. reported that the daily physical activity of AD patients is statistically significantly reduced [38], which may lead to muscle atrophy and decreased skin tension. This study found that skin elasticity in AD patients was lower than in the standard control group, which may be related to the aforementioned factors.

These skin physiological characteristics can serve as early biomarkers for predicting diseases with simple and quick examination methods. The findings can also aid in the early assessment and prediction of disease progression in AD. Healthcare providers can regularly monitor these skin features to understand changes in patient conditions and adjust treatment plans in a timely manner. If these features appear in patients, healthcare providers can conduct assessments and treatments early, improving treatment outcomes and patients’ quality of life.

Furthermore, we found a statistically significant positive correlation between carrying the ApoE E4 allele and favorable treatment outcomes. ApoE E4 is a common genetic variant in humans and a critical factor in cholesterol and lipid metabolism. Its presence is associated with an increased risk of AD, and studies suggest that it influences the pathogenesis of the disease by affecting lipid metabolism, leading to toxic accumulation and neuronal degeneration [39]. However, previous studies have seldom investigated the correlation between treatment efficacy and ApoE E4. Our study conducted a statistical analysis of CDR-SB changes in AD patients after six months of treatment and found that patients carrying ApoE E4 had a better response to treatment. Therefore, it is crucial to determine the ApoE genotype of Alzheimer’s patients, as it can improve risk assessment and diagnosis, and contribute to developing preventive measures and personalized treatment plans.

Fundamentally, the diagnosis of AD is based on a series of evaluations, including medical history, neuropsychological tests such as the MMSE, Montreal Cognitive Assessment (MoCA), CDR, imaging examinations, etc. Multiple biomarkers have been identified for assessing AD, including invasive techniques such as analyzing brain tissue for neurofibrillary tangles and amyloid plaques. However, these methods are not suitable for early detection in living patients. Analysis of cerebrospinal fluid (CSF) biomarkers such as β-amyloid (Aβ) and tau protein can provide useful diagnostic information. Aβ is the main component of amyloid plaques in AD [40], and its concentration in CSF usually decreases in AD patients. Tau protein is a structural protein in nerve cells, and its concentration in CSF usually increases in AD patients. Phosphorylation of tau protein (p-tau) is a marker of neuronal degeneration, and its concentration in CSF also typically increases in AD patients. In addition, some trace minerals such as Zn, Cu, Fe, and Al have been found to promote Aβ aggregation [31], and reactive oxygen and nitrogen species have been shown to promote neurodegeneration.

CSF sampling is a more invasive method for assessing AD, whereas peripheral blood biomarkers, such as plasma biomarkers, can be used as an alternative. Clusterin, a protein that can affect neuronal cell death, has been confirmed to be associated with the risk of developing AD. Neurospecific enolase (NSE), an enzyme highly expressed in neurons, has also been identified as a biomarker for AD, with increased levels in patients [31]. Decreased levels of high-density lipoproteins (HDL) in plasma have been associated with an increased risk of AD [41], indicating that cholesterol metabolism dysfunction may be a part of the pathogenesis of AD. Inflammatory mediators, such as IL-1, IL-6, and TNF-α, have been identified as potential biomarkers for evaluating AD patients [42], along with immune cell profiles and the complement cascade. Although Aβ and tau proteins can be detected in plasma, their concentrations are usually too low to serve as a reliable diagnostic criterion. Variations in AD patients’ skin or peripheral tissues have also been reported, such as the deposition of Aβ in the skin leading to fibroblast mutations [31].

Not all patients can undergo invasive tests, and non-invasive examinations are preferred to minimize additional harm to patients. Currently, non-invasive examinations such as Magnetic Resonance Imaging (MRI), Positron Emission Tomography (PET), and Single Photon Emission Computed Tomography (SPECT) are being used in clinical practice for the evaluation of AD [43]. Brain atrophy, gray matter volume changes, and white matter hyperintensities (WMHs) in MRI are possible changes in AD patients. In PET examinations, the deposition of β-amyloid and tau protein can be detected. The above-mentioned methods are known AD biomarkers, but non-invasive skin blood flow detection has rarely been proposed as an AD biomarker. We used a dynamic capillary microscope for nail folds and measured the skin microcirculation using laser Doppler flowmetry which can provide more objective measurements than previous methods and evaluations. Compared with other evaluation devices, skin physiology examination can provide information about the overall health status of patients, and it is non-invasive, safe, and economically applicable to large populations.

## 4. Materials and Methods

To achieve the study’s goal, the researchers recruited AD patients from the neurology department at the Kaohsiung Medical University Hospital from the period of 19 June 2012 to 18 June 2013. A total of 29 clinically diagnosed AD patients and 12 age-matched control subjects were recruited for this study. Because this was a pilot study to search for skin biomarkers in AD patients, the healthy controls were convenience samples from family members of the AD patients. Although this study had a small sample size, there was no selection bias. Future large-scale studies will be performed for further confirmation.

The criteria for AD and non-AD controls were as follows (Table 7):

The CDR performance was conducted according to the Washington University criteria, which can provide a pathologically validated accuracy of 93%. All participants had received comprehensive neuropsychological evaluations upon registration. The evaluations included the following measures.

### 4.1. The Mini-Mental State Examination (MMSE)

The MMSE ranging from 0 (worst) to 30 was used to describe general cognitive functioning.

### 4.2. Digit Span (Forward and Backward)

The Digit Span subtest of the WAIS-R in which the patient is required to repeat digits in forward and reverse order was used to measure basic attention.

### 4.3. Category Fluency (Animal)

In this test, the subject names as many animals as possible in one minute. The raw score is the number correctly named. This test was used to measure verbal production, semantic memory, and language.

### 4.4. Psychiatric Evaluations Using Neuropsychiatric Inventory (NPI)

The Neuropsychiatric Inventory (NPI) was used to interview caregivers, and screening questions were posed for each behavior. Caregivers were asked if the behavior represented a change from that exhibited by the patient before the onset of dementia and if it was present during the past month. If a positive response was obtained then the behavioral domain was explored with scripted questions focusing on specific features of the behavioral disturbance. Caregivers then rated the behaviors, with scores ranging from 1–4 for the frequency and 1–3 for the severity of each behavior (a composite score for each domain was the product of the frequency and severity subscores; maximum = 12).

### 4.5. Measurements of Skin Physiological Parameters

The measurements of skin hydration, pH, and elasticity were performed using the Multi Skin Test Center MC900^®^ (Courage and Khazaka, Cologne, Germany), while the assessment of skin blood flow was performed using a laser Doppler flowmeter (PeriFlux System 5000^®^; Perimed, Stockholm, Sweden). The measurements were carried out on reclining subjects after a 30-min acclimatization period at room temperature (20–22 °C) and 40–60% humidity.

### 4.6. Skin Hydration

The assessment of the hydration level of the skin surface (stratum corneum) was based on the Corneometer method, which measures the capacitance of a dielectric medium. Skin hydration was measured in relative units on a scale from 0–99. During measurement, the probe was applied to the skin surface with constant pressure.

### 4.7. Skin Surface pH Level

The assessment of skin surface pH at the test site was measured using a skin pH meter (PH 900, Courage and Khazaka, Cologne, Germany) based on an electrochemical method. The intraclass and interclass coefficient of variance was <10%. Skin pH was measured on a scale from 0–14.

### 4.8. Skin Elasticity

Skin elasticity assessments were performed on the left shoulders of all subjects by measuring the skin’s vertical deformation when pulled by a controlled vacuum into a circular aperture with an 8 mm diameter measuring probe.

### 4.9. Capillary Microscopy

Nailfold capillary assessment was performed using Capillary Microscopy (CAM1 capillary anemometer^®^, KK technology, London, UK). The nail fold of the fourth finger of the left hand was examined because this finger provides superior capillary visibility over all the fingers. Then, immersion oil was applied to the skin to maximize the transparency of the keratin layer of the epithelial cells. The collection of light magnification and a Doppler-shifted signal from capillary loops on nail fold established a real-time dynamic flow. Resting capillary blood cell velocity (mm/s) was measured. All subjects underwent high- (×200) microscopic examinations, with the total examination time ranging from 15 to 30 min. The number of capillaries was recorded, and the morphological changes as tortuous were counted. In the case of tortuous capillaries, the definition of the morphology was used for any crossing, meandering, or branching capillaries that is different from the regular hairpin-shaped capillaries.

### 4.10. Laser Doppler Flowmetry

Cutaneous microcirculatory assessments were carried out after 30 min of equilibration at a constant room temperature of 23 ± 1 °C. A laser Doppler flowmeter (PeriFlux^®^ 4001, Perimed AB^®^, Sweden) with a plastic holder was used for cutaneous blood flow measurements. The standard probe was fixed to the nail folds of the right and left fourth fingers for the simultaneous measurement of cutaneous blood flow. The recordings were continued for at least 30 min, and the measurements were repeated three times. The output signal of the laser Doppler was generated to more than 90% of the flow in subpapillary vessels. The blood flow was expressed as perfusion units (p.u.). Theoretically, the flow was determined by the product of the number of red blood cells moving in the measured volume (within the surface capillaries of the skin) and the mean velocity of these blood cells. Perisoft 5.10, the Perimed analysis program for PeriFlux^®^, was used to analyze the mean value of the perfusion unit within a defined period. The skin blood flow was recorded using a laser Doppler flowmeter (PF4001^®^) while the subjects remained in a resting position. The blood flow was recorded for 20 min in total. The intensity of the electric current was maintained at 200 At A during iontophoresis. The skin temperature was maintained at 30 °C throughout the experiment using a PeriTempTissue Heater, PF4005^®^ (Perimed, Sweden).

For each recruited participant, whether AD or non-AD, psychometric and clinical assessments of skin characteristics, including pH level and elasticity, as well as laser Doppler flowmetry were performed 6 months after the first evaluation.

### 4.11. ApoE Genotyping

For each AD patient, restriction enzyme isotyping of the apolipoprotein E (ApoE) allele was performed following a modification of the protocol developed by Pyrosequencer^®^ (http://www.pyrosequencing.com; accessed on 1 August 2012). Briefly, 10 ng of DNA was amplified in a 20 µL reaction volume in which dGTP was replaced by a mixture of 25% dGTP and 75% dITP to facilitate analysis of the GC-rich fragment. A 276bp fragment was generated using the following forward primer: AGA CGC GGG CAC GGC TGT, and the reverse Biotin-labeled primer was CTC GCG GAT GGC GCT GAG. Single-stranded DNA was prepared using streptavidin-coated beads, and the APOE gene variants at colons 112 and 158 were pyrosequenced using the following primers and dispensation order: SNP112 GAC ATG GAG GAC GTG, SNP158 CCG ATG ACC TGC AGA, and the dispensation order GCTGAGCTAGCGT.

### 4.12. Statistics

This study used GraphPad Prism 7.0 for statistical analysis. Other variables were expressed as frequency and percentage, and statistical analysis was performed using the chi-square test. Continuously variable items are presented as mean ± standard deviation (SD). The number of subjects included in this study was relatively small, which did not conform to the normal distribution as determined by the Shapiro-Wilk method. Therefore, comparisons between the two groups were performed using the Mann-Whitney nonparametric test. Correlation analyses employed the Spearman correlation test. In addition, we used logistic regression analysis to evaluate the impact and risk of related changes on MMSE improvement. A significance level of *p* < 0.05 was used in all analyses.

## 5. Conclusions

This study established skin as a non-invasive biomarker in AD patients. By applying skin biomarkers such as pH, elasticity, and nail fold capillary tortuosity to clinical practice, clinicians can better monitor changes in AD patients. We believe that physiological skin testing is a time-saving and non-invasive method that can effectively assist in evaluating changes in AD patients and serve as a reliable tool for monitoring disease progression, providing early diagnosis, and guiding treatment.

## Figures and Tables

**Table 1:** Clinical characteristics of AD patients and healthy controls.

	AD Patients (*n* = 29)	Healthy Controls (*n* = 12)	*p*-Value
Age (mean ± SD)	78.72 ± 7.32	75.33 ± 4.46	0.1447
Gender (number, %)			0.9533 ^#^
Male	7 (24.14%)	3 (25%)	
Female	22 (75.86%)	9 (75%)	
Education (year ± SD)	5.35 ± 3.50	6.91 ± 3.55	0.2001
Biophysiologic parameter (mean ± SD)			
PH	6.54 ± 1.36	5.90 ± 0.64	0.0294 *
Hydration	90.52 ± 12.59	73.50 ± 15.09	0.0012 **
Elasticity	48.48 ± 15.15	62.33 ± 7.09	0.0019 **
Capillary flow rate	0.35 ± 0.31	0.26 ± 0.08	0.8075
Total number of capillary	9.49 ± 3.47	10.17 ± 1.59	0.1210
Capillary tortuous number	3.83 ± 1.69	4.42 ± 2.19	0.5121
Percentage of tortuous capillary (%)	43.01 ± 20.07	44.38 ± 21.98	0.9657
Blood flow (perfusion unit)	0.23 ± 0.45	1.15 ± 0.72	0.5081

*p*-values were determined using the Mann-Whitney nonparametric test. ^#^ The *p*-values of the gender section were calculated using a chi-square test. * *p* < 0.05; ** *p* < 0.01.

**Table 2:** Correlations of two neurological evaluation methods: the Mini-Mental State Examination (MMSE) and Clinical Dementia Rating-Sum of the Boxes (CDR-SB) versus biophysiologic properties of AD patients at baseline.

Neurological Evaluation Methods	MMSE			CDR-SB		
	R	95% CI	*p*-value	R	95% CI	*p*-value
Biophysiological properties						
PH	0.10	−0.28–0.46	0.5918	−0.16	−0.50–0.23	0.4175
Hydration	0.33	−0.04–0.63	0.0726	−0.28	−0.59–0.11	0.1400
Elasticity	−0.14	−0.49–0.25	0.4679	0.19	−0.20–0.53	0.3280
Capillary flow rate	0.35	−0.03–0.64	0.0605	−0.06	−0.42–0.33	0.7688
Total number of capillaries	0.21	−0.18–0.55	0.2641	−0.29	−0.60–1.10	0.1334
Number of tortuous capillaries	−0.22	−0.55–0.17	0.2602	−0.19	−0.53–0.20	0.3221
Percentage of tortuous capillaries (%)	−0.49	−0.73–−0.14	0.0066 **	0.04	−0.34–0.41	0.8405
Blood flow (perfusion unit)	0.14	−0.33–0.56	0.5476	−0.02	−0.47–0.44	0.9303

*p*-values were determined using the Spearman correlation test; ** *p* < 0.01.

**Table 3:** Comparison of physiological parameters among responders and non-responders based on MMSE classification.

	Responder	Non-Responder	*p*-Value
Changes of MMSE	≧0	<0	
Case Number	14	15	
Biophysiological parameters (mean ± SD)			
PH	6.09 ± 1.45	6.97 ± 1.15	0.121
Hydration	92.64 ± 9.67	88.53 ± 14.88	0.615
Elasticity	50.57 ± 10.71	46.53 ± 18.55	0.555
Capillary flow rate	0.39 ± 0.37	0.32 ± 0.24	0.827
Total number of capillaries	8.64 ± 3.67	10.27 ± 3.20	0.098
Number of tortuous capillaries	4.21 ± 1.72	3.47 ± 1.64	0.211
Percentage of tortuous capillaries (%)	0.52 ± 0.22	0.35 ± 0.14	0.022 *
Blood flow (perfusion unit)	1.31 ± 0.40	1.15 ± 0.50	0.496

*p*-values were determined using the Mann-Whitney nonparametric test. * *p* < 0.05.

**Table 4:** Comparison of biophysiological parameters among responders and non-responders based on CDR-SB classification.

	Responder	Non-Responder	*p*-Value
Changes of CDR-SB	≦0	>0	
Case number	17	12	
Biophysiological parameters (mean ± SD)			
PH	6.67 ± 1.18	6.39 ± 1.61	0.991
Hydration	91.71 ± 11.46	88.83 ± 14.39	0.607
Elasticity	46.24 ± 13.95	51.67 ± 16.81	0.364
Capillary flow rate	0.36 ± 0.35	0.34 ± 0.24	0.882
Total number of capillaries	9.82 ± 3.61	9.0 ± 3.36	0.539
Number of tortuous capillaries	4.35 ± 1.66	3.08 ± 1.51	0.046 *
Percentage of tortuous capillaries (%)	0.48 ± 0.21	0.36 ± 0.17	0.168
Blood flow (perfusion unit)	1.24 ± 0.35	1.2 ± 0.63	0.918

*p*-values were determined using the Mann-Whitney nonparametric test. * *p* < 0.05.

**Table 5:** Logistic regression model for factors related to the improvement of MMSE (6 months, N = 29).

Covariate	Improvement of MMSE		
	*β*	OR (95% CI)	*p*-Value
Age	−0.016	0.984	0.752
Gender (male)	−2.159	0.115	0.064
Education level	−0.159	0.853	0.175
ApoE type (E4)	2.110	8.250	0.073
Hydration	0.028	1.029	0.382
PH	−0.630	0.533	0.105
Elasticity	0.019	1.019	0.470
Number of capillaries	−0.151	0.860	0.215
Capillary flow rate	0.756	2.130	0.555
Number of tortuous capillaries	0.281	1.324	0.234
Percentage of tortuous capillaries (%)	5.621	276.18	0.033 *
Blood flow (perfusion)	0.863	2.37	0.415

* *p* < 0.05.

**Table 6:** Logistic regression model for factors related to the improvement of CDR-SB (6 months, N = 29).

Covariate	Improvement of CDR-SB		
	*β*	OR (95% CI)	*p*-Value
Age	0.064	1.06	0.246
Gender (male)	−0.080	0.92	0.972
Education level	−0.014	0.99	0.898
ApoE type (E4)	2.565	13.00	0.031 *
Hydration	0.019	1.02	0.542
PH	0.177	1.19	0.540
Elasticity	−0.026	0.98	0.343
Number of capillaries	0.074	1.08	0.525
Capillary flow rate	0.195	1.22	0.877
Number of tortuous capillaries	0.530	1.70	0.056
Percentage of tortuous capillaries (%)	3.541	34.49	0.125
Blood flow (perfusion)	0.156	1.169	0.884

* *p* < 0.05.

**Table 7:** Inclusion criteria for AD and healthy controls.

Inclusion Criteria for AD Patients	Inclusion Criteria for Healthy Controls:
Patients with a clinical diagnosis of AD who fulfilled the DSM-IV criteria for dementia and NINCDS-ADRDA diagnostic criteria for probable AD.	a Subjects should be highly functioning individuals without subjective memory impairment or deficiency of cognitive function.
b Cognitive impairment demonstrated through neuropsychiatric testing, with an MMSE <27 and a clinical dementia rating (CDR).	b MMSE score ranging from 28–30 and CDR = 0.
c Generalized normal neurological examinations except impaired cognitive function.	c Not having a significant medical illness that might potentially interfere with cognitive functions, such as uncontrolled hypertension, diabetes, or metabolic or endocrinologic disorders.
d No structural brain abnormalities and volume loss of hippocampi, entorhinal cortex, or amygdale evidenced on MRI images.	d Excluding conditions that might interfere with the protein profiling, such as renal function impairment (with overt or microproteinuria).
e Memory and cognitive impairment not attributable to any medical conditions or medications.	e Able to provide informed consent and willing to join the present study.
f Exclusion of other neurodegenerative diseases.	
g The patients or their attorneys could understand the study’s objectives and provide informed consent.	

## Data Availability

The data presented in this study are available on request from the corresponding author. The data are not publicly available due to privacy.

## Supplementary Materials

The supporting information can be downloaded at: https://www.mdpi.com/article/10.3390/ijms24108478/s1.
