# Supplementary material for: Developing Biomarkers for the Skin: Biomarkers for the Diagnosis and Prediction of Treatment Outcomes of Alzheimer’s Disease
Source: Int J Mol Sci. 2023 May 9;24(10):8478. doi: 10.3390/ijms24108478 (PMC10218562; doi:10.3390/ijms24108478)
Supplement: Supplementary file 1 [file ijms-24-08478-s001.zip › ijms-2362649-supplementary.pdf]

**Supplementary Figure S1. Correlation between skin physiologic test and MMSE**

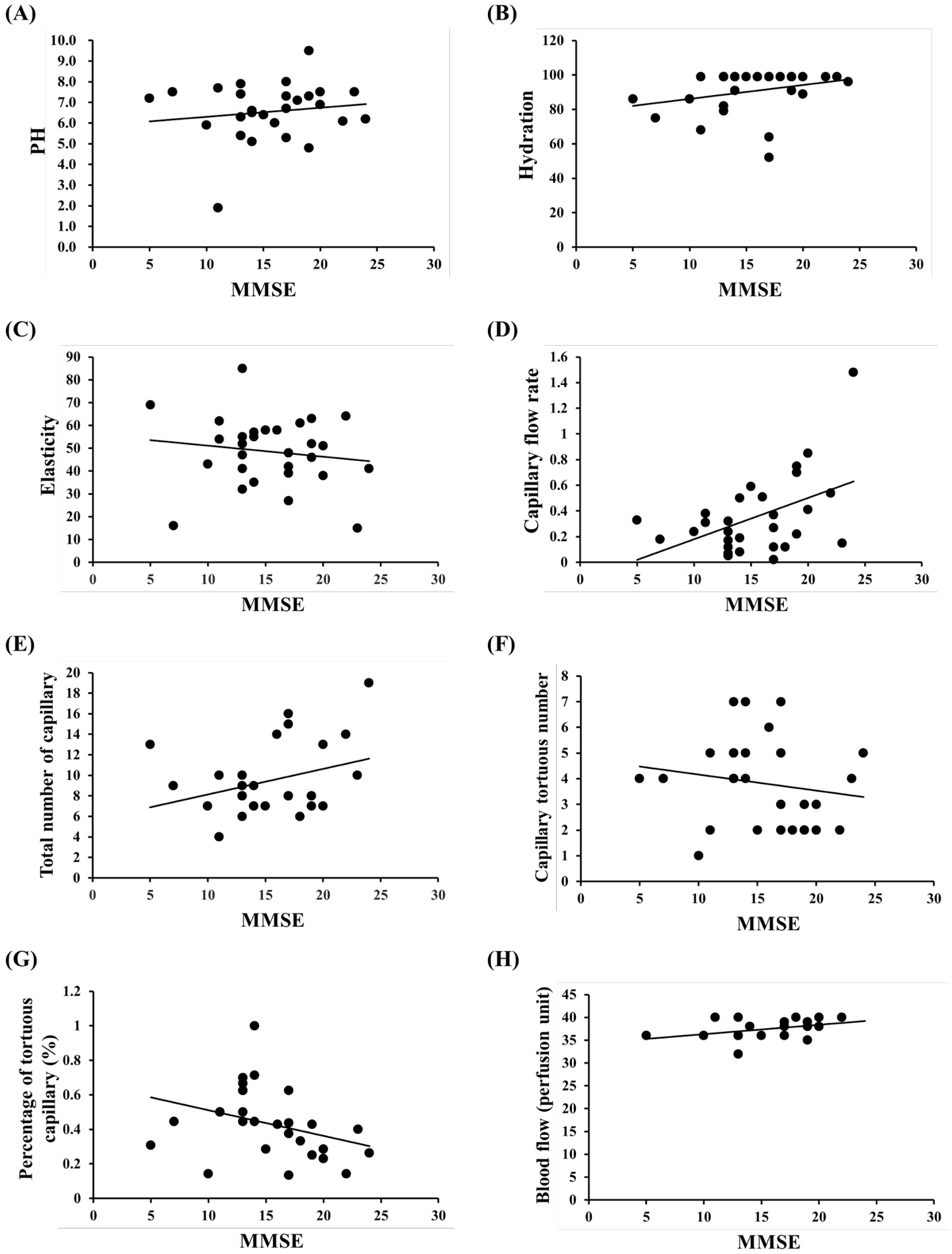


There is a statistically significant negative correlation (G) between the percentage of tortuous capillaries and the MMSE score (with an R-value of −0.49, 95% confidence interval of −0.73 to −0.14, and a *p*-value of 0.0066). All other skin physiologic parameters (A to F, and H) are not correlated with the MMSE score. *p*-values were determined using the Spearman correlation test

## Supplementary Figure S2. Correlation between skin physiologic test and CDR-SB score

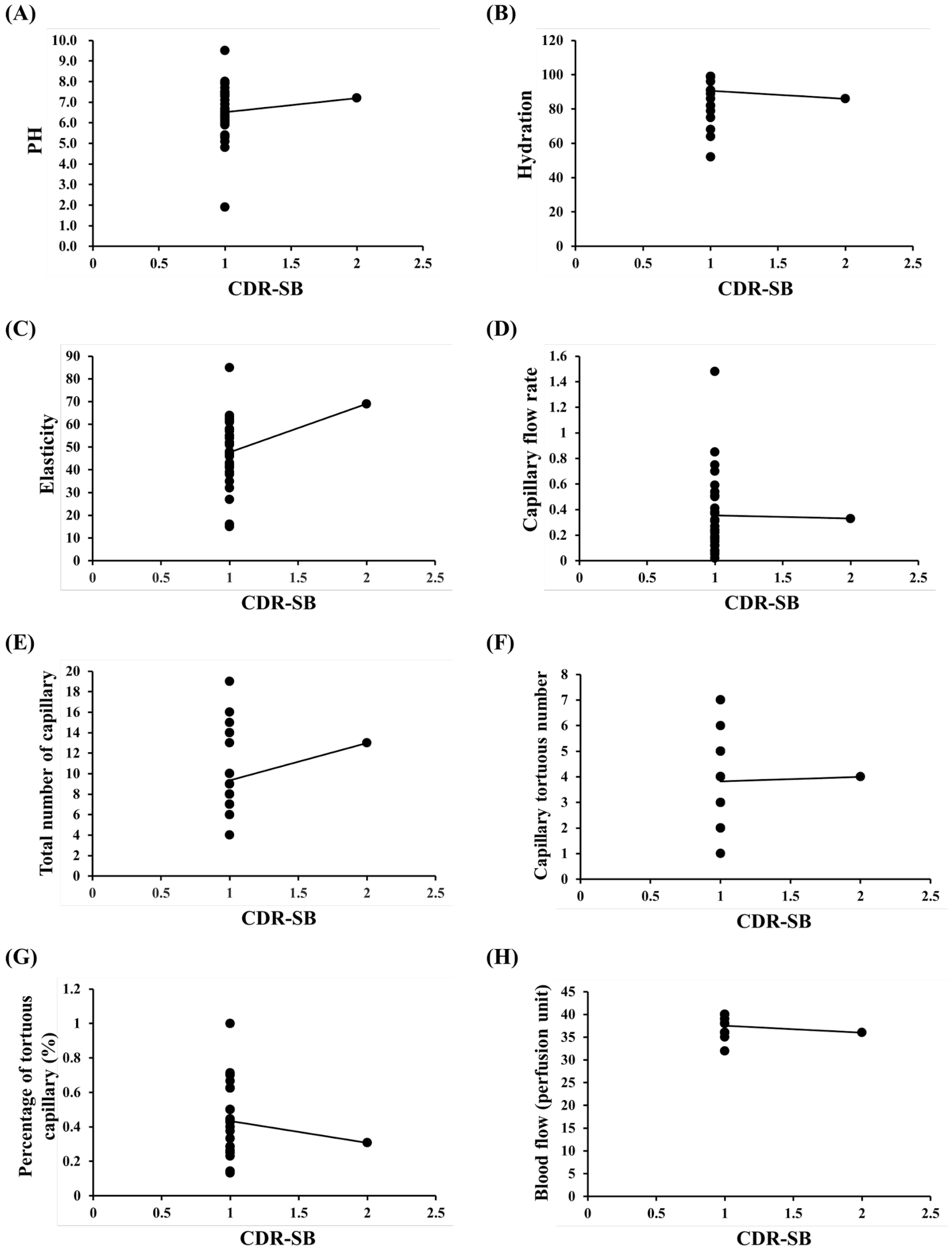


This study did not show a statistically significant correlation between CDR-SB scores and skin physiological values (A to H).
